# Supplementary material for: The topography of frequency and time representation in primate auditory cortices
Source: eLife. 2015 Jan 15;4:e03256. doi: 10.7554/eLife.03256 (PMC4398946; doi:10.7554/eLife.03256)
Supplement: Supplementary file 1. — Directions and relative orientations of amplitude modulation rate and frequency gradients in selected auditory field (based on best rate/frequency maps). Main gradient directions (relative to anterior-posterior axis) and the resulting relative angle (α) between the orientations of the amplitude modulation rate (Rate) and spectral frequency (Frequency) gradients in auditory fields A1, R and CL are listed for two hemispheres (L, R) in three animals (M1-3). Additionally, R2 values, p-values and number of data points (n) from the respective 2D regression analysis are included. * No defined gradient direction due to single best frequency in field. DOI: http://dx.doi.org/10.7554/eLife.03256.008 [file elife03256s001.docx]

| **Animal** | **Hemis-** | **Field** | **Rel. angle (α)** | Frequency | R^2^ | p-value | Rate | R^2^ | p-value | n |
| --- | --- | --- | --- | --- | --- | --- | --- | --- | --- | --- |
|  | **phere** |  | [degrees] | [degrees] |  |  | [degrees] |  |  | (vertices) |
|  |  |  |  |  |  |  |  |  |  |  |
|  |  |  |  |  |  |  |  |  |  |  |
| **M1** | **L** | **A1** | 164 | 168 | 0.794 | <1E-16 | 3 | 0.314 | 2.34E-07 | 84 |
|  | **R** |  | 75 | 165 | 0.677 | <1E-16 | 90 | 0.806 | <1E-16 | 101 |
| **M2** | **L** |  | 49 | 176 | 0.878 | <1E-16 | 127 | 0.735 | <1E-16 | 154 |
|  | **R** |  | 109 | 172 | 0.84 | <1E-16 | 79 | 0.517 | <1E-16 | 103 |
| **M3** | **L** |  | 142 | 167 | 0.672 | <1E-16 | 51 | 0.649 | <1E-16 | 156 |
|  | **R** |  | 111 | 167 | 0.813 | <1E-16 | 57 | 0.342 | 2.26E-08 | 87 |
|  |  |  |  |  |  |  |  |  |  |  |
| **Average** |  |  | **108.5** | **169.2** | **0.78** |  | **67.8** | **0.56** |  | **114.2** |
| **StdDev** |  |  | **42.1** | **4.1** | **0.09** |  | **41.8** | **0.20** |  | **32.5** |
|  |  |  |  |  |  |  |  |  |  |  |
|  |  |  |  |  |  |  |  |  |  |  |
| **M1** | **L** | **R** | 65 | 80 | 0.560 | 6.66E-16 | 145 | 0.243 | 7.30E-06 | 88 |
|  | **R** |  | 123 | 17 | 0.316 | 2.74E-07 | 140 | 0.806 | <1E-16 | 107 |
| **M2** | **L** |  | 165 | 28 | 0.779 | <1E-16 | 138 | 0.241 | 1.19E-06 | 102 |
|  | **R** |  | 27 | 28 | 0.574 | 2.22E-16 | 0 | 0.191 | 7.06E-07 | 93 |
| **M3** | **L** |  | 161 | 8 | 0.397 | 9.55E-10 | 168 | 0.238 | 1.13E-05 | 87 |
|  | **R** |  | 131 | 2 | 0.427 | 3.42E-09 | 133 | 0.362 | 1.48E-07 | 73 |
|  |  |  |  |  |  |  |  |  |  |  |
| **Average** |  |  | **112.0** | **27.2** | **0.51** |  | **120.7** | **0.35** |  | **91.7** |
| **StdDev** |  |  | **55.1** | **27.9** | **0.17** |  | **60.4** | **0.23** |  | **12.1** |
|  |  |  |  |  |  |  |  |  |  |  |
|  |  |  |  |  |  |  |  |  |  |  |
| **M1** | **L** | **CL** | 157 | 50 | 0.887 | 6.00E-15 | 107 | 0.324 | 2.80E-03 | 33 |
|  | **R** |  | 153 | 164 | 0.134 | 1.45E-05 | 42 | 0.522 | 2.72E-07 | 44 |
| **M2** | **L** |  | 81 | 64 | 0.611 | 4.18E-08 | 145 | 0.514 | 1.63E-06 | 40 |
|  | **R** |  | -* | - | - | - | 85 | 0.233 | 7.77E-04 | 58 |
| **M3** | **L** |  | -* | - | - | - | 7 | 0.911 | <1E-16 | 48 |
|  | **R** |  | 5 | 13 | 0.238 | 5.00E-03 | 179 | 0.642 | <1E-16 | 42 |
|  |  |  |  |  |  |  |  |  |  |  |
| **Average** |  |  | **99.1** | **72.8** | **0.47** |  | **94.2** | **0.52** |  | **44.2** |
| **StdDev** |  |  | **71.7** | **64.5** | **0.35** |  | **63.8** | **0.24** |  | **8.4** |
